# Mixed methods evaluation of a digital resource to build students’ skills in **AS**sessing cardiovascular risk, **MO**tivating change, and **SUS**taining a healthier lifestyle in themselves and others- **ASMOSUS**: a study protocol

**DOI:** 10.1186/s12912-025-02923-2

**Published:** 2025-03-10

**Authors:** L. Creighton, G. Caughers, G. Thompson, G. Mitchell, B. Forrest, S. McHale, N. McKenna, B. J. Rice, A. Smart, S. Gilhooly, P. Gordon-Wright, D. Fraser, S. Gray, Y. Eom, E. Kennedy, C. McLaughlin, J. McMahon, C. L. Hanson, L. Neubeck, D. Fitzsimons

**Affiliations:** 1 School of Nursing and Midwifery, Medical Biology Centre, Queen’s University Belfast, 97 Lisburn Road, Belfast, BT9 7BL Northern Ireland, UK; 2 Centre for Cardiovascular Health, Edinburgh Napier University, Sighthill Campus, Edinburgh, EH11 4DN Scotland, UK

**Keywords:** Nursing students, Cardiovascular disease risk, Healthy lifestyles, Health promotion, Self-efficacy, Cardiovascular risk management, Digital resource, Education

## Abstract

**Background:**

Cardiovascular disease (CVD) is a prevalent cause of morbidity and mortality globally. Nurses and nursing students are in an optimum role to assess, manage and promote lifestyle changes associated with CVD risk. Patients and service users are more likely to adopt these changes if the person delivering the information embodies this lifestyle themselves. Literature suggests that nurses and nursing students show detrimental behaviours in association with smoking, obesity, nutrition, physical inactivity and alcohol. It is therefore essential to address CVD risk factors, management and lifestyle promotion early on in a healthcare professionals’ career- ideally the university delivering their nursing program. This aligns with the Nursing and Midwifery Council curricula in the United Kingdom (UK) on the topic of public health and health promotion. Although already taught there is a gap between knowledge and adoption of healthy lifestyle behaviours. This is potentially resolved through consolidating self-efficacy in nursing students and their ability to apply theory to practice.

**Methods:**

This study will evaluate a digital educational resource: ASMOSUS. This resource was co-designed with nursing students, academic and clinical staff to provide the skills to assess CVD risk, motivate change and encourage adoption of a healthy lifestyle in themselves and others. All nursing students will receive the ASMOSUS digital resource as part of their routine teaching, followed by either a 90-minute face to face tutor-led class or via a live online platform such as Microsoft Teams to consolidate skills with their peers. A mixed-methods study will be carried out in two phases. Phase one will use two questionnaires to investigate student knowledge on CVD risk and self-efficacy, using a pre-post test design. Phase two will explore the experience of the students in using the resource and the impact on their skills and self-efficacy using focus groups.

**Discussion:**

This study has the potential to engage nursing students as the health professionals of the future in the early adoption of the knowledge and skills in CVD risk assessment, management and promotion of a healthy lifestyle. This will inform not only the health and wellbeing of nursing students themselves but translate into role modelling for patients and optimal patient care.

**Supplementary Information:**

The online version contains supplementary material available at 10.1186/s12912-025-02923-2.

## Background

Cardiovascular diseases (CVD) are the leading cause of death globally [1]. Early identification and management of CVD risk factors are essential in the prevention of the potential complications that can arise from this non-communicable disease. Within the healthcare workforce, nurses are optimally situated to identify CVD risk factors and support people with adopting healthier lifestyles, thus reducing mortality and morbidity [2]. Patient and public compliance with advice issued by nurses is influenced by the health-related behaviours of these healthcare professionals [3]. However, literature suggests that the health of student and registered nurses in the United Kingdom (UK) is sub-optimal. Detrimental levels of obesity [4, 5], smoking [5, 6] alcohol consumption [5, 6] physical activity [5, 6] and dietary intake [6, 7] are evidenced in these populations.

Unfavourable lifestyle behaviours identified in nursing students [5, 6] have the potential to diminish their capacity to influence the adoption of cardioprotective lifestyles in others [7]. The Nursing and Midwifery Council (NMC) is the governing body that informs the undergraduate nursing curricula content across the UK but also who students must declare themselves fit to practice to on completion of their nursing program. One of the NMC proficiency standards suggests that nurses should have ‘an ability to manage their own personal health and wellbeing and make a significant contribution to health promotion.’ [8]. Health promotion is a taught element of the nursing curricula, and it is therefore unlikely that these unfavourable lifestyle behaviours can be explained by a lack of knowledge. It may be related to self-efficacy, an individual’s confidence with executing a specific behaviour [9] in influencing the ability of nursing students to implement healthy lifestyle behaviours. A correlation between health-promoting behaviour and enhanced self-efficacy has been demonstrated in nursing students [10].

It is known that student application of theory into practice can be challenging [11], with a recent study in the UK concluding that educational interventions that improve the implementation of health-related behaviours for student nurses are warranted [12]. A European study [13] found that qualified cardiac nurses felt under prepared for their role with more education required on CVD risk factor management. This is supported in a further study [14] that cites a barrier to CVD prevention in patient care is a deficit in professionals’ knowledge. It is important to narrow this knowledge gap as it appears to widen through years of practice. To address this requirement in educational improvement, the study team across Queen’s University Belfast (QUB) and Edinburgh Napier University (ENU) have co-designed and developed, with nursing students a digital resource called ASMOSUS (**AS**sessing cardiovascular risk, **MO**tivating change, and **SUS**taining a healthier lifestyle in themselves and others), which aims to teach students knowledge and skills in CVD risk assessment, management and cardioprotective lifestyle implementation.

## Aim

The aim of this study is to carry out a mixed-methods evaluation of the impact of the ASMOSUS digital resource on the knowledge, attitudes, ability of CVD risk management and healthy lifestyle implementation of first year undergraduate nursing students at QUB and ENU.

The study objectives are to:

- Investigate the effect of the ASMOSUS resource on students’ knowledge and self-efficacy with CVD risk management and healthy lifestyle promotion, and the suitability of the resource.
- Explore the students’ experiences with the ASMOSUS resource and their perspective regarding its’ impact on their skills, confidence and ability with CVD risk management and implementing or promoting healthy lifestyles.

### Digital ASMOSUS resource

The digital resource has been developed following co-design methodology [15, 16] in a 2-stage process. Stage 1 consisted of two on-line workshops, using an iterative approach to content development. The content was informed by nursing students (*n* = 3) QUB and (*n* = 4) ENU and facilitated by academic staff representing both universities. Workshop 1 was an exploration of knowledge, learning requirements and relevant educational topics. Workshop 2 developed ideas of preferred platform, layout and formatting of the resource.

The information from the workshops was collated and consolidated by the study team to draft the content and structure for the ASMOSUS resource. Essential topics were identified as lifestyle behaviours and CVD risk, CVD risk assessment and Motivational Interviewing. Resource development was carried out by expert digital resource engineers Focus Games Ltd., to last around 30 min, provide self-directed learning and have animations, quizzes and simulated scenarios. The self-directed, digital resource will be supported by the application of learning workshops face-to-face on campus or via a live online platform such as Microsoft Teams, lasting 90 min. These will be tutor-led and allow nursing students to undertake motivational interviewing scenarios with their peers (Supplementary file 1).

## Methods

The study will use an explanatory sequential mixed methods design to investigate the effect of using the ASMOSUS resource as part of routine teaching for cohorts of year one nursing students across two universities (one in Northern Ireland and one in Scotland). The study objectives will be achieved in 2 phases. Phase 1 a pre-post questionnaire design and phase 2 online focus groups.

All year 1 undergraduate nursing students, across all fields (adult, learning disability, mental health, children and young people) from both QUB and ENU who receive the ASMOSUS resource via routine teaching will be eligible for recruitment. The approximate yearly intake of both universities is 1350 students. Year 1 undergraduate nursing students are the chosen population as their awareness and behaviours with regards to cardiovascular disease risk and awareness are similar to the general population characteristics. Commencing this awareness of cardiovascular disease and motivational interviewing early in their nursing programme allows for modifiable lifestyle behaviours that are not favourable to be adapted sooner.

A sample of at least 491 students is required to achieve a representative sample for Phase 1 data collection (*n* = 249 QUB and *n* = 242 ENU). Sample size was determined through a power analysis calculation using Raosoft http://www.raosoft.com/samplesize.html, based on a total available sample size of 1350, at a confidence level of 95% and an accepted margin of error 5%. Thus ensuring that the study has a sufficient likelihood of detecting statistically significant differences between the pre and post-test responses for the Attitudes and Beliefs about Cardiovascular Disease (ABCD) Risk questionnaire [17] and the ASMOSUS Impact Questionnaire (Supplementary file 2).

In Phase 2, recruitment sample aim for four focus groups with five participants per group is *n* = 20 (*n* = 10 QUB and *n* = 10 ENU). The choice of *n* = 20 students for focus group aligns with qualitative research principles that emphasize data saturation, wherein new insights cease to emerge as the sample size increases, found to be within four to eight focus groups [18]. With a focused and homogeneous population such as nursing students, a smaller sample size can often be sufficient to capture the range of perspectives and experiences. Additionally, the focus on qualitative insights in this phase aligns well with the exploratory nature of the study’s second objective. The use of convenience sampling is pragmatic, considering that all year one nursing students will have access to the ASMOSUS resource as part of routine teaching. This approach facilitates access to participants and streamlines recruitment efforts, contributing to feasibility. Moreover, the study’s cross-university design, involving nursing students from institutions in Northern Ireland and Scotland, enhances the generalizability of the findings. This diversity of participants strengthens the study’s ability to draw broader conclusions and insights that could potentially be applicable to a wider range of nursing education contexts.

### Recruitment phase 1

Prior to the routine delivery of the ASMOSUS resource to the cohort, all students will be contacted via email by a the year lead who will act as a gatekeeper (a person not involved in this research study) to inform them of the evaluation. The email will contain a link to the Participant Information Sheet (PIS) and details of the research team contact. The resource will be embedded in an appropriate teaching module alongside a link to an online e-consent form, followed on from which are two questionnaires to complete pre- self-directed learning and application of learning workshop and 3 questionnaires to complete post. This will be asynchronous to the resource itself and collected during the semester where the nursing students are in the university setting.

### Recruitment phase 2

Approximately 4 weeks following engagement with the resource an email will be sent to all students in the nursing cohort by the year lead to inform them about online focus groups that will take place via Microsoft teams, whilst students remain in the university setting.

The email will contain a link for a PIS and contact for the research team. Interested students will register to attend via an ‘Eventbrite’ link on the teaching module. Online consent will be obtained prior to the online focus group by a facilitator, using a link in the ‘chat’ function in Microsoft Teams. The focus groups will only be conducted with those students who provide informed consent.

### Data collection instruments- phase 1

Participating students will be invited to complete two questionnaires; the ABCD Risk questionnaire [17], which is a validated evaluation of knowledge of CVD risk and an ASMOSUS Impact Questionnaire a bespoke questionnaire developed by the research team.

The ABCD risk 65- item questionnaire is composed of four scales; perceived risk (Cronbachs’s Alpha 0.85), perceived benefits, intention to change behaviour (Cronbach’s Alpha 0.82) and nutrition (Cronbach’s Alpha 0.60) [17]. Responses to the questionnaire are via a self-report Likert scale with five options; strongly agree, agree, neutral, disagree and strongly disagree.

The ASMOSUS Impact 16-item questionnaire will assess the effect of the resource content on students’ knowledge and self-efficacy with CVD risk management and healthy lifestyle promotion. The questionnaire was a bespoke design by the research team with alignment to the digital educational resource content and the outcome of self-efficacy. Responses are by a self-reported 5 item Likert scale; strongly agree, agree, neutral, disagree and strongly disagree. Face validity was undertaken with a group of 30 year one undergraduate nursing students separate to the study recruitment population [19, 20]. Using a subjective assessment of the following factors; relevance, formatting, readability, clarity and the appropriateness for the intended audience, feedback was recorded using an online form that was anonymous, minor changes to wording only was recommended to add clarity. A pilot study will also be undertaken prior to phase 1 data collection where the planned psychometric tests will be utilised.

Demographic details such as field of nursing degree, age, gender, ethnicity, highest education and partial postcode for socio-economic status, will be collected pre-test to set the findings in context. The System Usability Scale [21] post-test 10-item questionnaire will be used as a validated method of assessing the suitability of the resource (Cronbach’s Alpha 0.85), self-reported responses are via a Likert scale with 5-items: strongly agree, agree, neutral, disagree and strongly disagree. Time to complete all questionnaires will be five to ten minutes for participants. Student number will be the only identifier, this will be removed post paired analysis and held confidentially until then, followed by deletion.

### Data collection process- phase 2

Focus groups will be facilitated by academic staff in the respective universities, both on the project team, with one ideally who has not delivered the ASMOSUS workshops. The focus group will take place on-line via Microsoft teams and will last approximately 30–45 min. A focus group question guide (Supplementary file 3) will be followed and aims to explore the experience of utilising the resource and the transferable learning to their own and others lifestyle, determination of CVD risk factors and management. The focus groups will be audio recorded to enable transcription and analysis. For methodological rigour phase 2 of the study will adhere to the consolidated criteria for reporting qualitative research (COREQ) checklist [22] (Supplementary file 4).

### Analysis

In phase 1 the quantitative data will be analysed using SPSS V28. Descriptive statistics will be used to profile the sample and paired t-tests will be carried out on the remaining data to determine if there is a statistical significance between the pre and post-test questionnaires for both the ABCD risk questionnaire [17] and the ASMOSUS Impact Questionnaire. The System Usability Scale [21] will be analysed separately as it was completed post intervention only.

The focus group audio recordings in phase 2 will be transcribed verbatim by an external service and identifying information removed. Qualitative data will be analysed using the stages of the framework method of analysis; familiarisation, identifying a thematic framework, indexing, charting, mapping and interpretation [23]. To enhance rigour a process of member checking will be implemented, to ensure the analysis adequately reflects the participants account [24]. Qualitative data analysis will be uploaded to NVivo management software.

The quantitative and qualitative data will be integrated to achieve a triangulation of findings, which may generate a multidimensional understanding of the impact of the ASMOSUS resource [25]. The reporting of the integrated data will comply with the ‘Good Reporting of a Mixed Methods Study’ (GRAMMS) framework [26].

## Discussion

CVD is a leading concern for the health of the general population. Primary prevention is a key factor to address this issue and nurses as healthcare professionals are in a position to address this issue. Sub-optimal lifestyle behaviours in healthcare professionals [4–7] can have a detrimental impact on patients and services users adopting changes into their own lifestyle [2], with the literature suggesting that nursing students are among those professionals. As evidenced in the UK nursing curricula [8] nursing students are empowered with the knowledge but often this does not match with their embodied behaviours. Gaining confidence in motivating change in themselves and others is key to applying CVD risk knowledge to themselves and others. The research team is hopeful that the use of the ASMOSUS educational digital resource, followed by a tutor-led workshop allowing nursing students to consolidate their learning and practice skills in Motivational interviewing, will be a step towards self-efficacy in CVD risk awareness and prevention.

## Electronic supplementary material

Below is the link to the electronic supplementary material.

Supplementary Material 1

Supplementary Material 2

Supplementary Material 3

Supplementary Material 4

## Data Availability

No datasets were generated or analysed during the current study.

## Abbreviations

CVD: Cardiovascular disease
UK: United Kingdom
QUB: Queens University Belfast
ENU: Edinburgh Napier University
NMC: Nursing and midwifery council
ASMOSUS: ASsessing cardiovascular risk, MOtivating change, and SUStaining a healthier lifestyle in themselves and others
ABCD risk questionnaire: Attitudes and beliefs about cardiovascular disease risk questionnaire
PIS: Participant information sheet
COREQ: consolidated criteria for reporting qualitative research
GRAMMS: Good reporting of a mixed methods study
