# Supplementary material for: Mixed methods evaluation of a digital resource to build students’ skills in ASsessing cardiovascular risk, MOtivating change, and SUStaining a healthier lifestyle in themselves and others- ASMOSUS: a study protocol
Source: BMC Nurs. 2025 Mar 10;24:264. doi: 10.1186/s12912-025-02923-2 (PMC11895151; doi:10.1186/s12912-025-02923-2)
Supplement: Supplementary file 1 — Supplementary Material 1 [file 12912_2025_2923_MOESM1_ESM.docx]

**ASMOSUS Impact Questionnaire (Queens’ University Belfast and Edinburgh Napier University)**

*This questionnaire will ask about your level of agreement with statements related to your knowledge and confidence with cardiovascular disease (CVD) risk management and healthy lifestyle promotion.*

**1.** I am confident I have the knowledge to identify CVD risk in my own lifestyle behaviors

| **Strongly disagree** | **Disagree** | **Neutral** | **Agree** | **Strongly agree** |
| --- | --- | --- | --- | --- |
| ð | ð | ð | ð | ð |

**2.** I am confident I have the knowledge to discuss CVD risk with others

| **Strongly disagree** | **Disagree** | **Neutral** | **Agree** | **Strongly agree** |
| --- | --- | --- | --- | --- |
| ð | ð | ð | ð | ð |

**3.** I know how to approach a CVD risk assessment for myself and others

| **Strongly disagree** | **Disagree** | **Neutral** | **Agree** | **Strongly agree** |
| --- | --- | --- | --- | --- |
| ð | ð | ð | ð | ð |

**4.** I am confident I can communicate CVD risk to others

| **Strongly disagree** | **Disagree** | **Neutral** | **Agree** | **Strongly agree** |
| --- | --- | --- | --- | --- |
| ð | ð | ð | ð | ð |

**5.** I understand what is meant by motivational interviewing

| **Strongly disagree** | **Disagree** | **Neutral** | **Agree** | **Strongly agree** |
| --- | --- | --- | --- | --- |
| ð | ð | ð | ð | ð |

**6.** I understand the main processes of motivational interviewing

| **Strongly disagree** | **Disagree** | **Neutral** | **Agree** | **Strongly agree** |
| --- | --- | --- | --- | --- |
| ð | ð | ð | ð | ð |

**7**. I am confident I can undertake a motivational interview

| **Strongly disagree** | **Disagree** | **Neutral** | **Agree** | **Strongly agree** |
| --- | --- | --- | --- | --- |
| ð | ð | ð | ð | ð |

**8.** I understand the different stages of willingness to change a lifestyle behaviour

| **Strongly disagree** | **Disagree** | **Neutral** | **Agree** | **Strongly agree** |
| --- | --- | --- | --- | --- |
| ð | ð | ð | ð | ð |

**9**. I am confident with supporting lifestyle change in people who are at different stages of willingness to change (*e.g.,* contemplation, maintenance, and relapse)

| **Strongly disagree** | **Disagree** | **Neutral** | **Agree** | **Strongly agree** |
| --- | --- | --- | --- | --- |
| ð | ð | ð | ð | ð |

**10**. I have the confidence to use a motivational interview to help someone reduce or stop smoking

| **Strongly disagree** | **Disagree** | **Neutral** | **Agree** | **Strongly agree** |
| --- | --- | --- | --- | --- |
| ð | ð | ð | ð | ð |

**11**. I have the confidence to use a motivational interview to help someone increase physical activity

| **Strongly disagree** | **Disagree** | **Neutral** | **Agree** | **Strongly agree** |
| --- | --- | --- | --- | --- |
| ð | ð | ð | ð | ð |

**12**. I have the confidence to use a motivational interview to help someone improve his / her diet

| **Strongly disagree** | **Disagree** | **Neutral** | **Agree** | **Strongly agree** |
| --- | --- | --- | --- | --- |
| ð | ð | ð | ð | ð |

**13**. I have the confidence to use a motivational interview to help someone reduce his / her stress

| **Strongly disagree** | **Disagree** | **Neutral** | **Agree** | **Strongly agree** |
| --- | --- | --- | --- | --- |
| ð | ð | ð | ð | ð |

**14**. I have the confidence to use a motivational interview to help someone reduce his / her alcohol intake

| **Strongly disagree** | **Disagree** | **Neutral** | **Agree** | **Strongly agree** |
| --- | --- | --- | --- | --- |
| ð | ð | ð | ð | ð |

**15**. I would recommend motivational interviewing for promoting healthy lifestyle changes in the future

| **Strongly disagree** | **Disagree** | **Neutral** | **Agree** | **Strongly agree** |
| --- | --- | --- | --- | --- |
| ð | ð | ð | ð | ð |

**16**. I am likely to use motivational interviewing to support people with healthy lifestyle changes in the future

| **Strongly disagree** | **Disagree** | **Neutral** | **Agree** | **Strongly agree** |
| --- | --- | --- | --- | --- |
| ð | ð | ð | ð | ð |
