# Supplementary material for: Mixed methods evaluation of a digital resource to build students’ skills in ASsessing cardiovascular risk, MOtivating change, and SUStaining a healthier lifestyle in themselves and others- ASMOSUS: a study protocol
Source: BMC Nurs. 2025 Mar 10;24:264. doi: 10.1186/s12912-025-02923-2 (PMC11895151; doi:10.1186/s12912-025-02923-2)
Supplement: Supplementary file 2 — Supplementary Material 2 [file 12912_2025_2923_MOESM2_ESM.docx]

**Focus Group Guide**

**Title of Project:**

ASMOSUS: Co-creation of a digital resource to build students’ skills in ASsessing cardiovascular risk, MOtivating change, and SUStaining a healthier lifestyle in themselves and others.

**[All focus groups will be moderated by two members of the research team; one of whom will not have previously taught the students]**

***Introduction***

The purpose of this focus group is to discuss your experience with the ASMOSUS resource and your perspective on its impact on your skills, confidence, and ability with cardiovascular disease (CVD) risk management and implementing / promoting healthy lifestyles going forward. If any participant wishes to stop or withdraw from the focus group at any time, please let me know and the discussion will be stopped.

***Topic Areas:***

**What were your overall impressions of the ASMOSUS resource?**

- What did you think about the resource in general?
- What did you enjoy most about the resource?
- What did you enjoy least about the resource?

**What was the impact of the ASMOSUS resource?**

- Did this resource influence your understanding of CVD risk and making healthy lifestyle changes?
- Do you feel more confident about managing CVD risk and making healthy lifestyle changes after completing the resource?
- Do you feel more competent with assessing CVD risk and promoting healthy lifestyle changes in others?

**Did the ASMOSUS resource help you to make healthy lifestyle changes in yourself and / or others?**

- Did you use the learning to help yourself or others recognise CVD risk?
- Did the resource enable you to identify or change any lifestyle behaviours in yourself or others?

**What are the limitations of the ASMOSUS resource?**

- Were there any challenges when using the ASMOSUS resource?
- How might the resource be improved for students in the future?
- Were there any difficulties with applying your learning from the resource?
- Were there any important areas of learning that the resource did not cover?

***Close***

Thank you for your time and effort in this focus group; everything discussed has been very helpful. Before the focus group ends, I would like to ask if anyone has any other thoughts or feelings you would like to share to help me better understand your experience or is there anything you would like me to go back to?
