# Supplementary material for: Mixed methods evaluation of a digital resource to build students’ skills in ASsessing cardiovascular risk, MOtivating change, and SUStaining a healthier lifestyle in themselves and others- ASMOSUS: a study protocol
Source: BMC Nurs. 2025 Mar 10;24:264. doi: 10.1186/s12912-025-02923-2 (PMC11895151; doi:10.1186/s12912-025-02923-2)
Supplement: Supplementary file 3 — Supplementary Material 3 [file 12912_2025_2923_MOESM3_ESM.docx]

**Supplementary File**

Consolidated criteria for reporting qualitative studies (COREQ): 32-item checklist.

| **No** | **Item** | **Guide questions/description** |
| --- | --- | --- |
| **Domain 1: Research team and reflexivity** |  |  |
| Personal Characteristics |  |  |
| 1. | Interviewer/facilitator | All interviews will be facilitated by both GM and LC |
| 2. | Credentials | GM has a PhD, LC is a lecturer and PhD researcher and both have expertise in qualitative research methods and qualitative data collection (including focus-group interviewing). |
| 3. | Occupation | GM is a Reader at the university and LC is a lecturer and PhD researcher. |
| 4. | Gender | GM is male and LC is female. |
| 5. | Experience and training | GM is a registered nurse with considerable experience in higher education and qualitative methodology. LC is a registered nurse, lecturer and PhD researcher with experience in higher education. |
| Relationship with participants |  |  |
| 6. | Relationship established | GM & LC are educators at Queen’s University Belfast and teach on the Undergraduate Nursing Programme where students were recruited. |
| 7. | Participant knowledge of the interviewer | Participants will be aware of who is conducting the focus groups as these are detailed in the information sheet and consent forms. |
| 8. | Interviewer characteristics | GM is a registered nurse and lectures in undergraduate nursing. GM also has a strong awareness in the context (e.g., design, implementation and evaluation of serious educational games in higher education). LC shares expertise in education of year one nursing students and has collaborated with GM on similar research. |
| **Domain 2: study design** |  |  |
| Theoretical framework |  |  |
| 9. | Methodological orientation and Theory | Mixed methods evaluation comprised of pre/post-test questionnaire followed by thematic analysis of focus group data. |
| Participant selection |  |  |
| 10. | Sampling | Convenience sampling of year one nursing students at the university undertaking BSc Professional Nursing Degree. |
| 11. | Method of approach | Participants were approached by a gatekeeper (Director of Education) that was not associated with the study. |
| 12. | Sample size | 249 participants in phase one and 10 participants in phase two (qualitative aspect). |
| 13. | Non-participation | Participants will be reminded that participating in this research will not affect their course grade.This is noted in the information sheet and within the consent form. |
| Setting |  |  |
| 14. | Setting of data collection | Focus group data will be collected via online meetings (MS Teams). Data collection will take place during the student’s own time. |
| 15. | Presence of non-participants | There will be no non-participants present during the focus group interviews. |
| 16. | Description of sample | Participants are year one undergraduate nursing students from the university. |
| Data collection |  |  |
| 17. | Interview guide | The interview guide was designed by academics involved in the study and reviewed by the student co-design Participants. The guide seeks to explore how the educational resource influences student nursing practice and self-efficacy after engagement. |
| 18. | Repeat interviews | No repeated interviews were carried out. |
| 19. | Audio/visual recording | All focus group data will be audio-recorded. |
| 20. | Field notes | No field notes will be collected during focus group interviews. |
| 21. | Duration | Focus group interviews will last approximately 30 minutes. |
| 22. | Data saturation | Data saturation will be estimated to be achieved in this study with the participation of 10 nursing students in focus groups at QUB site. |
| 23. | Transcripts returned | All participants have the option of reviewing their focus group transcript as noted in the information sheet, consent form and at the conclusion of the focus group interview. |
| **Domain 3: analysis and findings** |  |  |
| Data analysis |  |  |
| 24. | Number of data coders | Qualitative data analysis, using thematic analysis (Braun and Clarke), will carried out by LC. |
| 25. | Description of the coding tree | All academic authors will actively participate in the design, data collection, analysis, and interpretation of the study, ensuring comprehensive collaboration and shared contributions to the research process. |
| 26. | Derivation of themes | The authors will use thematic analysis to analyse themes. This will be led by LC initially and involve all team members in the advanced stages. |
| 27. | Software | The authors will use NVivo Version 14 to manage the data. |
| 28. | Participant checking | Member checking will be carried out to ensure accuracy of transcription and emergent themes. |
| Reporting |  |  |
| 29. | Quotations presented | Direct quotations will be presented from participants whilst confidentiality maintained. |
| 30. | Data and findings consistent | The authors will ensure there is concordance between the data and findings that are presented. All authors will be involved. |
| 31. | Clarity of major themes | The authors will report all major themes as identified in this research. |
| 32. | Clarity of minor themes | The authors will report on any minor themes or deviant cases within their presentation of results if these emerge. |
