# Supplementary material for: Mixed methods evaluation of a digital resource to build students’ skills in ASsessing cardiovascular risk, MOtivating change, and SUStaining a healthier lifestyle in themselves and others- ASMOSUS: a study protocol
Source: BMC Nurs. 2025 Mar 10;24:264. doi: 10.1186/s12912-025-02923-2 (PMC11895151; doi:10.1186/s12912-025-02923-2)
Supplement: Supplementary file 4 — Supplementary Material 4 [file 12912_2025_2923_MOESM4_ESM.docx]

Tutor-led ASMOSUS digital educational resource programme outline

**Programme duration total 90 minutes**

1. Recap of the resource content using a Power Point presentation- separated into the 3 modular blocks of the resource:

Cardiovascular Disease Risk

Cardiovascular Disease Risk Awareness

Motivational Interviewing

Each section will be delivered using a standardised slide deck and participating students will have the opportunity to ask any questions during a pause post each modular block.

**Time allocation 45 mins**

2) Application of knowledge

Participating students will have the opportunity to carry out the peer role play scenarios generated by the ASMOSUS resource. Working in groups of three they will all have the opportunity to lead a motivational interview.

**Time allocation 30 minutes** (Timer on ASMOSUS resource 10 minutes allocated per scenario)

3) Group debrief on motivational interviewing scenarios

Discussion of the scenarios undertaken with the overall group and opportunity to ask any questions.

**Time allocation 15 minutes**
